# The importance of screening for trauma symptoms in children and adolescents

**DOI:** 10.1192/j.eurpsy.2025.1108

**Published:** 2025-08-26

**Authors:** T. Bollain Muñoz, I. Oliveira Amat, R. Crespo Rubio, J. Gomez Puebla, T. Gomez Escribano, J. M. Martinez Avila

**Affiliations:** 1 Psychiatry, HGUGM, Madrid, Spain

## Abstract

**Introduction:**

Children and adolescents frequently encounter a range of adverse childhood experiences (ACEs), which encompass various forms of adversity such as abuse, neglect, and household dysfunction. These experiences can have profound and lasting effects on an individual’s health and well-being. Alarmingly, nearly three out of four children—approximately 300 million aged 2 to 4 years—are subjected to physical punishment and/or psychological violence by parents and caregivers. Moreover, statistics indicate that one in five women and one in thirteen men were sexually abused during their childhood (ages 0-17). Despite the widespread prevalence of these experiences, trauma in children often goes unrecognized. The nature of trauma can make it challenging for both the child and caregivers to identify and articulate trauma-related symptoms. Children may struggle to understand or express their experiences, and caregivers might misinterpret or overlook these signs, leading to underreporting and a lack of timely intervention.

**Objectives:**

Experiencing adverse events during childhood or adolescence is particularly concerning because it can significantly disrupt normal developmental trajectories, affecting physical, emotional, and cognitive growth. During these formative years, the brain is highly plastic and sensitive to environmental influences, making it especially vulnerable to the effects of trauma and stress. Such exposure can result in long-term consequences, including a heightened risk of developing mental health disorders, behavioral issues, and challenges in academic and social settings.

In this context, early identification of children and adolescents who have faced adverse experiences is crucial. By providing appropriate support and resources early on, it is possible to foster resilience and promote more positive growth despite the challenges posed by early adversity.

**Methods:**

Using tools like the Child PTSD Symptom Scale (CPSS), a widely recognized self-report instrument designed to assess the severity of post-traumatic stress disorder (PTSD) symptoms in children and adolescents aged 8 to 18, can be especially effective for identifying and evaluating the impact of trauma exposure in young individuals and facilitating early intervention.

**Results:**

Research published by the National Institute of Mental Health (NIMH) indicates that early identification through screening can lead to timely interventions, significantly reducing the psychological harm associated with trauma exposure.

**Conclusions:**

Research strongly supports the effectiveness of screening for trauma symptoms in children and adolescents, emphasizing its critical role in early detection, timely intervention, and the prevention of long-term negative outcomes. This proactive approach not only addresses the immediate psychological impact of trauma but also contributes to improved long-term well-being and quality of life for those affected.

**Disclosure of Interest:**

None Declared

